# Comparison between standard and reduced volume radiotherapy in bladder preservation trimodality protocol for muscle-invasive bladder cancer patients

**DOI:** 10.3332/ecancer.2016.682

**Published:** 2016-10-26

**Authors:** Waleed Arafat, Azza Darwish, George E Naoum, Wael Sameh, Gamal El Husseiny, Fathy Abd-El-Gawad, Mostafa Samir

**Affiliations:** 1 Oncology Department, Faculty of Medicine, Alexandria University, Egypt; 2 Alexandria Comprehensive Cancer Centre, Alexandria, Egypt; 3 University of Alabama at Birmingham, 1720 2nd Ave S, Birmingham, AL 35233, USA; 4 Urology Department, Faculty of Medicine, Alexandria University, Egypt

**Keywords:** bladder cancer, lymph nodes, radiotherapy, trimodality protocol

## Abstract

**Aim:**

Our aim is to compare the toxicity, pelvic nodal relapse, and overall survival of whole bladder irradiation only to the standard technique of whole pelvis irradiation followed by bladder boost in patients with muscle-invasive bladder carcinoma undergoing bladder preservation protocol.

**Material and method:**

A total of 60 patients with transitional cell carcinoma, stage T2-3, N0, M0 bladder cancer were subjected to maximal transurethral resection bladder tumour (TURB). Then, the patients were randomised into two groups: group I (30 patients) to receive whole pelvis radiotherapy 44 Gy followed by 20 Gy bladder boost. While group II (30 patients) were randomised to receive whole bladder radiotherapy alone for a total dose of 64 Gy. In both groups, concomitant cisplatin and paclitaxel were given weekly throughout the whole course of radiotherapy where conventional 2 Gy/fraction were used. Additionally, four cycles of adjuvant cisplatin and paclitaxel were given after the end of the chemoradiotherapy induction course.

**Results:**

The first assessment after the induction chemoradiotherapy showed that complete response was achieved in 73.3% of patients in group I and 76.7% of the patients in group II. After a median follow-up of 2 years, regional relapse occurred in 7.1% of patients in group I and 10.3% in group II. (*p* = 1). Distant metastases were detected in 17.9% of patient in group I and 13.8% in group II (*p* = 0.73). The 2-year disease-free survival was 60% in group I and 63.3% in group II (*p* = 0.79). The whole 2-year overall survival was 75% in group I and 79.3% in group II (*p* = 0.689). Radiation gastrointestinal (GI) acute toxicity was higher in group I than in group II (*p* = 0.001), while late GI radiation toxicity was comparable in both groups.

**Conclusion:**

Treating the bladder only, without elective pelvic nodal irradiation, did not compromise pelvic control rate, but significantly decreased the acute radiation toxicity.

## Introduction

Bladder cancer ranks ninth in worldwide cancer incidence. It is the seventh most common cancer in males and the 17th most common cancer in females [1]. Males are 2.5–5 times more likely to develop this malignancy than females [2].

Globally, the incidence of bladder cancer varies significantly, with Egypt, Western Europe and North America having the highest incidence rates and Asian countries the lowest rates [3]. Incidence of bladder cancer increases with age and peaks in the sixth and seventh decades of life [4].

Radical cystectomy with urinary diversion is still the standard of care for muscle-invasive bladder cancer in many institutions, particularly in the USA [5]. However, radical cystectomy may affect quality of life, as patients may be at risk of several early and late morbidities that include haemorrhage, infection, urinary leaks, pelvic lymphocele, intestinal obstruction and peritonitis [6]. Even the construction of a neobladder after cystecomy cannot substitute for the patient’s original bladder, owing to late complications. These include pyelonephritis, strictures, calculi, fistula formation, continence problems and the need for intermittent self-catheterisation, necessitating long-term follow-up in these patients [7]. Besides these several problematic complications of radical cystectomy, this surgery results in only 40–60% 5-year overall survival, and despite a high local control attained by this approach, 20–30% of patients may develop local relapse with or without metastatic spread [8]. It is also to be noted that lymph node positivity can decrease the disease-free survival and overall survival of these patients undergoing radical cystectomy [9]. The same study by Ugurlu *et al*. also showed that these positive lymph nodes are the only factor affecting survival and even their removal did not improve the survival of these patients. On the other hand, a study by Wright *et al*. showed that an increased number of lymph nodes removed at the time of cystectomy was associated with improved survival in patients with lymph node-positive bladder cancer [22].

However, over the past 20 years, multimodality organ-sparing treatment has become the standard of care for many malignancies; therefore, the question has arisen as to whether primary cystectomy in the treatment of bladder cancer could also be replaced by an organ-sparing treatment option [10].

Sufficient data now exist from many international prospective studies to demonstrate that the survival results from bladder preservation protocols, using trimodality treatment strategies involving maximal TUR, radiation therapy and chemotherapy, compare favourably to radical cystectomy [11, 23, 33]. However, radiotherapy, the principal part of trimodality therapy, showed wide variation as regards prescription doses and treatment volumes worldwide [12].

Despite the current advances in imaging, computerised planning and three-dimensional conformal radiation, acute, and long-term toxicities are still be considered when embarking upon bladder preservation protocols. Many RTOG trials have been faced by GI and pelvic toxicity problems as the induction part of radiotherapy is usually delivered to the whole pelvis followed by a bladder boost [13–15]. Taking into consideration, the toxicity of this radiation planning along with the controversial role of positive lymph node removal on survival, we hypothesised that omitting pelvic nodal irradiation might improve toxicity outcomes allowing these patients protocol continuation and hence better survival outcome.

In this study, we report our institute’s experience, aiming to estimate the local control, survival, and toxicity of a novel radiotherapy planning technique including only the whole bladder rather than whole pelvis followed by bladder boost in patients undergoing bladder preservation protocols.

## Methodology

This study included 60 patients with muscle-invasive bladder tumour (T2–T3) with no clinical metastases.

Patients who were eligible to enter the study had to fulfil the following criteria: (A) Operable patients with histologically proven transitional cell carcinoma of the bladder (muscle invasive stage T2 and T3); (B) Age ≥ 18 years; (C) WHO performance status ≤ 2 at the start of treatment (Appendix I). (D) Patients must be considered able to tolerate systemic chemotherapy combined with pelvic radiotherapy. € Adequately functioning bladder. (F) Treatment began within 8 weeks following TUR. (G) Normal organ functions through: (i) Adequate bone marrow function as indicated by “Hemoglobin ≥ 10 mg/dl”, “WBC ≥ 4000/ml”, “Absolute neutrophilic count ≥ 1800/ml”, and “Platelets ≥ 100,000/mm3”. (ii) Adequate renal function as indicated by “Serum creatinine < 1.5 × the upper limit of normal” and “Creatinine clearance ≥ 60 ml/min”. (iii) Adequate liver function as indicated by “Serum bilirubin <2 mg/dl” and “ALT, AST < 2 × upper limit of normal”. (H) Participation in the trial was according to the patients’ preference after full description of the advantages and disadvantages of definitive chemoradiotherapy of the bladder and radical cystectomy. (I) Patient’s written informed consent.

Patients with any of the following were not eligible for the trial: (A) Evidence of tumour-related hydronephrosis. (B) Evidence of distant metastasis or lymph node metastasis. (C) Uncontrolled systemic disease that would preclude the patient from the study. (D) Pregnancy. (E) Inflammatory bowel disease. (F) Previous pelvic radiotherapy or systemic chemotherapy. (G) Concurrent usage of drugs that have potential nephrotoxicity or ototoxicity. (H) Patient judged not to be a candidate for radical cystectomy. (I) Other malignancy within the previous two years (other than adequately treated BCC of the skin or adequately treated *in situ* carcinoma of the cervix).

**Figure 1.**
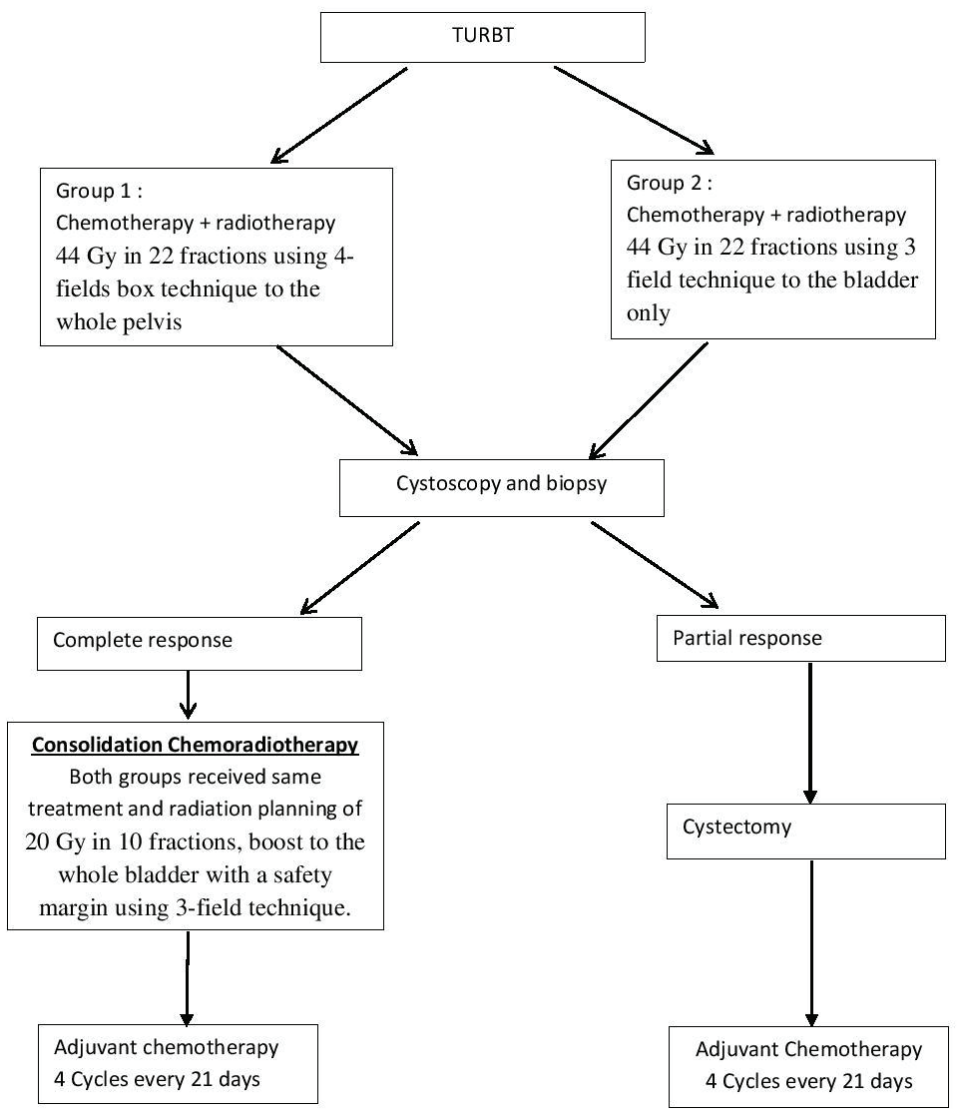
General study design.

After obtaining IRB (institutional review board) approval 60 patients were randomised into two treatment groups. Both groups received the same general protocol design as depicted in Figure 1, but they differed in the radiotherapy planning and volume in induction phase only. Where group I received the standard radiotherapy technique of bladder cancer: 44 Gy in 22 fractions using 4-field box technique to the whole pelvis, and group II received 44 Gy in 22 fractions using 3-field technique to the bladder only. In the consolidation phase, both groups received the same radiation planning of 20 Gy in 10 fractions, boost to the whole bladder with a safety margin using 3-field technique. Concomitant cisplatin and paclitaxel were given weekly throughout the radiotherapy course, and four cycles cisplatin, paclitaxel were given as adjuvant therapy following chemoradiation.

## Radiotherapy technique included the following:

A. Localisation, simulation, and immobilisation

Radiotherapy began within 8 weeks following TURBT, patients were instructed to void before simulation, in order to empty the bladder, Foley catheter was inserted into the patient shortly after voiding. The post-void urine residual was measured, and this volume was replaced by an equal volume of bladder contrast plus an additional amount of contrast and air. It is to be noted that the amount of liquid in the bladder did not exceed 30 ml, because it is desirable to treat the patients with their bladder empty in order to minimise the day-to-day mobility of the bladder. Contrast in the rectum was not used. The rectum should be as empty as possible before simulation. Patients were positioned supine and immobilised by vacuum bags. The laser localiser system was used to put three radioopaque marks for position reproducibility. These fixed marks positions were the reference used for repositioning of the patients prior to treatment delivery. CT tomogram was generated first and reviewed prior to acquiring the planning scan to ensure that the patient alignment was correct. Then, the patients were scanned from the bottom of the ischial tuberosities to 3 cm above the bladder dome or the bottom of L5 whichever was higher. A scan of interval 5 mm was used.

B. Target volume and dose specificaiton

Patients in group I received. Whole pelvis with a high-energy linear accelerator with 15 MV photons using 4-field box technique for a 44 Gy in 22 fractions, one fraction per day, five fractions per week. The planning target volume included: the upper border at L5–S1, lower border: just below the obturator foramen, the lateral margin of the anteroposterior field at 1.5 to 2 cm laterally beyond the widest point of pelvic cavity, the anterior border of the lateral field at 2 cm anterior to the bladder boundary, the posterior border of the lateral field at 2.5 cm beyond the bladder or any visible tumour mass. The lymph nodes were contoured so that:

- Presacral nodes: Extends from L5–S1 to the top of S3 and includes 1–1.5 cm of tissue anterior to the sacrum and between the vessel contours
- Iliac nodes: Generated by expanding the iliac vessel contours by 7 mm in the anterior, posterior, and lateral dimensions, but not the superior or inferior dimensions.
- Obturator nodes: Encompass 1 cm width of tissue medial to the obturator internus muscles extending from the anterior border of the ilium to the posterior border of the ilium. Contoured started superiorly at the inferior border of the iliac vessel contours and extending inferiorly to the top of the pubic symphysis

The bladder boost included the whole bladder plus any extravesical tumour extension plus anisotropic margins (2 cm at the superior and anterior walls and 1.5 cm at all other walls) to a dose of 20 Gy in 10 fractions, one fraction per day, five fractions per week. This was given by linear accelerator with 6 MV and 15 MV photons using 3-field technique depending on specific characteristics of the individual patient.

While patients in group II received: whole bladder radiotherapy including the whole bladder plus any extravesical tumour extension plus anisotropic margins (2 cm at the superior and anterior walls and 1.5 cm at all other walls) to a total dose of 64 Gy; (44 Gy in 22 fractions during induction and 20 Gy in 10 fractions during consolidation, one fraction per day, five fractions per week). This was given by linear accelerator with 6 MV and 15 MV photons using 3-field technique depending on specific characteristics of the individual patient.

In both radiotherapy groups, treatment was given by three-dimensional radiotherapy.

C. Critical structures

The maximum dose to the femoral heads should be less than 45 Gy. Fifty per cent of the rectum should receive less than 55 Gy. The rectum volume was defined on CT from the anus (at the level of ischial tuberosities) for a length of 15 cm or to the rectosigmoid flexure.

## Chemotherapy included the following:

A. Concurrent chemotherapy that was given through radiotherapy where: paclitaxel 50 mg/m2 administered as one-hour infusion weekly during the course of radiotherapy and cisplatin 15 mg/m2 administered as one-hour infusion the first three days of each week. Taking into consideration that radiotherapy started one hour after the infusion chemotherapy was completed.

B. Adjuvant chemotherapy (starting 4 weeks following the post consolidation endoscopic evaluation or 8 weeks following surgery) through paclitaxel 175 mg/m2 (3-hour infusion) and cisplatin 75 mg/m2 (2-hour infusion). Taking into consideration that therapy was repeated every 21 days for 4 cycles.

### Statistical analysis

The analysis of the results of this study was carried out by the intention-to-treat principles, which indicate that all randomised patients should be included in the primary analysis of the results. Exclusion of patients from analysis due to early death, disease progression or any other than eligibility was not done as this can severely distort the results. All analysis was done using Statistical Package for the Social Sciences (SPSS), version 15.

### Survival analysis

The overall survival (OAS) was calculated from the date of diagnosis to the date of death. Disease free survival (DFS) was calculated from the date of diagnosis to the date of treatment failure (recurrence of disease or distant metastasis). Kaplan–Meier life table was used to study OAS and DFS.

### Tests of significance

T-test, analysis of variance (ANOVA) and Chi-square were used to study significance differences between variables.

## Results

This study was carried out on sixty patients from our Clinical Oncology Department. All patients in the current study fulfilled the eligibility criteria and their characteristics are depicted in Table 1. The median follow-up period was 2 years.

### 1. Initial response

The first assessment after the induction chemoradiotherapy showed that there was no statistically significant difference (*P* = 0.135) as regards the response to induction chemoradiotherapy as shown in Table 2. Forty-five patients (22 in group I, 23 in group II) achieved complete response and proceeded to consolidation chemoradiotherapy. Whereas 15 patients (8 in group I, 7 in group II) had incomplete response after the initial assessment. Out of these 15 patients, nine patients only of the incomplete responders had undergone radical cystectomy. One patient (in group II) was prepared for radical cystectomy, but surgery was delayed several times to correct his general condition, then he died before undergoing the surgery. Five patients refused cystectomy (3 in group I and 2 in group II), of these five patients, three were lost follow-up (2 in group I and 1 in group II) and the remaining two patients insisted to proceed consolidation chemoradiotherapy (one in each group) even after discussing with them the disadvantages of their choice.

### 2. Disease-free survival

**Figure 2.**
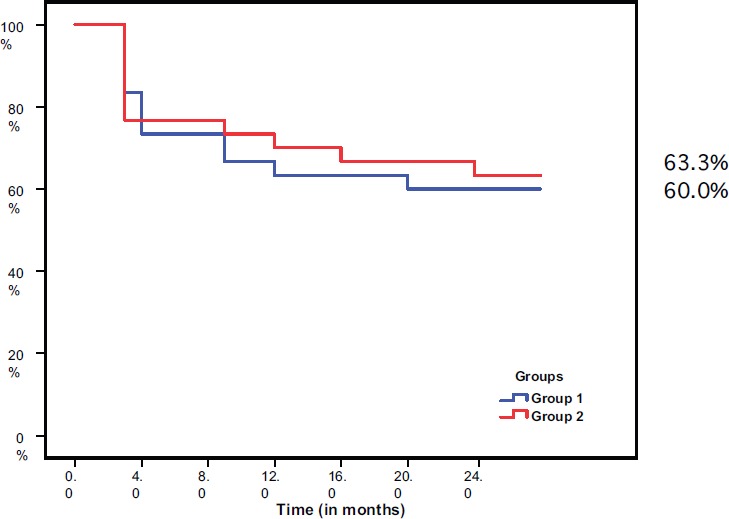
Disease-free survival in both groups.

After 24 months of follow-up, the disease free survival (DFS) was 60% in group I and 63.3% in group II. There was no statistically significant difference between the two groups (*P* = 0.791) (Figure 2)

### 3. Regional relapse and distant metastasis

A. Two patients (7.1%) patients in group I and three patients (10.3%) in group II developed regional relapse during the period of follow-up. None of these patients had isolated regional relapse, as one of these patients (in group I) had associated local invasive relapse while in the remaining four patients (one in group I and three in group II) distant metastasis were also detected. There was no significant difference between both groups as regards the regional relapse (*P* = 1).

B. Five patients (17.9%) in group I and four patients (13.8%) in group II developed distant metastasis with a median time of 13 months. Lung and bone were the most common sites of distant metastasis in both groups. There was no statistical significant difference between both groups as regards the incidence of distant metastasis (*P* = 0.730) (Table 3)

### 4. Acute toxicity

Acute toxicity was collected from patients and then scored according to RTOG/EORTC classification. The analysis was based on the evaluation of the maximum toxicity score throughout treatment for each patient.

a. Acute radiation toxicity during the induction phase

Acute genitourinary toxicity, in both groups, was mostly in the form of frequency, nocturia and dysuria in 29 patients in group I and 28 patients in group II with no statistical significance (*P* = 1.0) as shown in (Table 4). Acute GIT toxicity, in both groups, was mostly in the form of diarrhoea and rectal pain in 28 patients in group I and only five patients in group II with a statistical significance (*P* < 0.0001).

b. Acute toxicity during the consolidation phase

None of the patients in both groups had grade-3 or grade-4 urinary toxicity during the consolidation phase with 21 patients in group I and 20 patients in group II with no statistical significance (*P* = 0.666). Also, all forms of GIT toxicity in both groups was grade 1 or grade 2, with three patients in group I and two patients in group II (*P* = 0.666) during the consolidation phase as shown in Table 5.

### 5. Toxicity during the adjuvant chemotherapy

Both groups were comparable as regards the haematological and non-haematological toxicities during the adjuvant chemotherapy with no significant difference. However this data is not shown.

### 6. Late toxicity

The worst toxicity that occurred or persisted after the third month from the end of consolidation chemoradiotherapy was considered as late toxicity. However, all cases who developed late urinary or GIT toxicity were grade 1 or 2 as shown in Table 6. There was no statistical significance between the two groups in both urinary and GIT toxicity with (*P* = 0.792) for the first and (*P* = 0.609) for the second.

### 7. Overall survival

**Figure 3.**
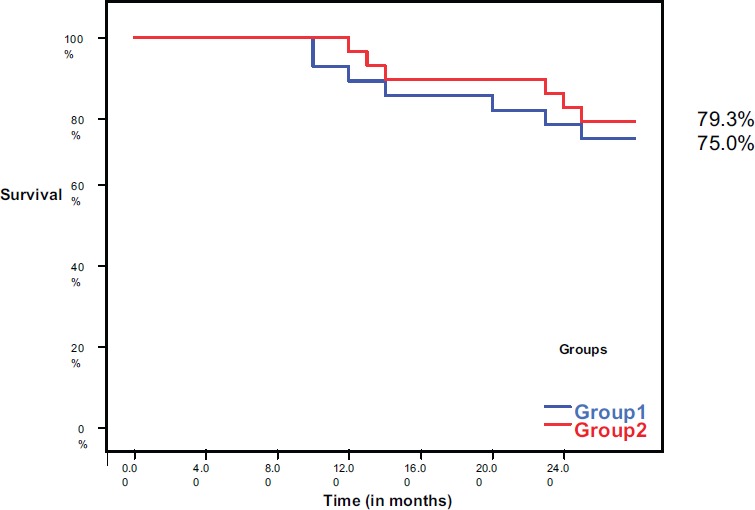
Overall survival in both groups.

During the 2 years of follow-up, 13 patients (22.8%) had died; there are seven patients (25%) in group I and six patients (20.7%) in group II. The 2-year survival was 75% in group I, while it was 79.3% in group II with no significant difference between both groups (*P* = 0.760) (Figure 3).

**Figure 4.**
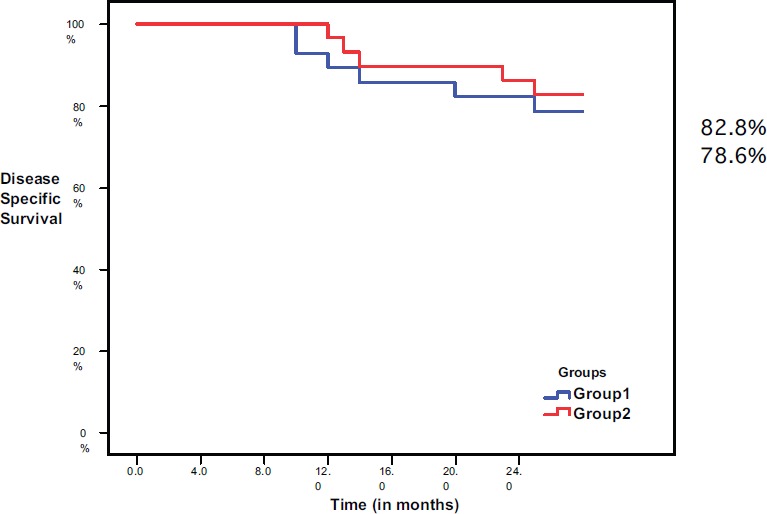
Disease-specific survival in both groups.

The disease-specific survival, at the end of follow-up period, was 78.6% in group I and 82.8% in group II, with no significant difference (*P* = 0.689) (Figure 4).

## Discussion

Bladder cancer is the 9th most common cancer diagnosis worldwide, with more than 330,000 new cases each year and more than 130,000 deaths per year, with an estimated male:female ratio of 3.8%:1.0% [24].

Worldwide it is estimated that 4,300,000 new cases occur each year, with the highest incidence in industrialised countries and areas where infection with the parasite Schistosoma haematobium is endemic. The majority of non-schistosomiasis-related cases are transitional cell carcinoma where squamous cell carcinoma is related to schistosomiasis [25].

In Egypt, bladder cancer has been the most common cancer during the past 50 years [26–28]. Egypt’s world-standardised bladder cancer incidence was representing approximately 30,000 new cases each year [29]. Interestingly, the most common histopathological type of bladder cancer in Egypt has been SCC, constituting from 59% to 81% of reported bladder cancers between 1960 and 1980 [28, 30]. Oncologists and pathologists in Egypt have suggested that there is a changing ratio of SCC:TCC types of bladder cancer over the past 10–15 years. Previous research has reported a significant decrease in SCC in Egypt, although the overall bladder cancer incidence in Egypt has remained steady due to an increase in TCC over the past 30 years [31]. One study examining 2778 cases in the Nile Delta Region, Metropolitan Cairo area, and the South of Egypt reported that, in1980, 22% of Egyptian bladder cancer cases were diagnosed with TCC and 78% were diagnosed with SCC; however, by 2005, that ratio was nearly the opposite with 73% of bladder cancers diagnosed as TCC and 28% diagnosed as SCC [31]. In this study, Most of the patients in the present study had associated bilharziasis, 76.7% in group I and 63.3% in group II. This was consistent with the results reported by Sabaa *et al*. [32] where 78.8% of patients had associated bilharziasis.

The trimodality bladder-sparing protocol is considered an optimum approach for bladder cancer. In bladder-sparing protocols, radiotherapy is the principal part of the local treatment with wide variation of the prescription doses and treatment volumes.

The most commonly used technique is to treat the whole pelvis including the pelvic lymph nodes to a dose of 40–50 Gy (with conventional fractionation). Followed by an additional boost dose, approximately 20 Gy with conventional fractionation, to the whole bladder [12].

Another technique is to deposit the full dose to a volume containing the bladder with margins only, [16] this is based on the concept that elective irradiation of pelvic lymph nodes will result in a greater gastrointestinal toxicity than bladder only radiation technique [17]. Additionally, the increasing integration of systemic chemotherapy into radiation protocols may be adequate to treat micrometastatic disease in pelvic lymph nodes and neutralise any potential benefit of elective pelvic nodal irradiation [18].

Despite all these encouraging results, the acute and late toxicity of this protocol remains obstacles that oncologists need to overcome. This study was designed to assess the role and effect of lymph node omission as a novel radiation planning technique attempting to achieve better results.

The first assessment after the induction chemoradiotherapy showed that complete response was achieved in 73.3% of patients in group I and 76.7% of the patients in group II. This is consistent with the complete response rate of 60–80%, that is observed in most of bladder preservation protocols, after the induction therapy [19]. Hagan *et al*., [11] in the RTOG 97-06 trial, had reported that 74% of the patients had achieved complete response after induction chemoradiotherapy. Kaufman *et al*., [23] in the RTOG 99-06 trial, found that 81% of the patients had complete response after induction therapy, also Lin *et al*. [20] had found that 76.7% of the patients achieved complete response. On the other hand, Shipley *et al*. [21] had reported that only 63% of the patients had complete response after the induction phase.

In the current study, we followed our patients for 24 months. Three patients (2 in group I, 1 in group II) were lost follow-up after showing incomplete response at the end of the induction chemoradiotherapy phase. Follow-up of the 45 patients (22 in group I, 23 in group II) who achieved complete response and proceeded to consolidation chemoradiotherapy revealed that, 80% of these patients (77.3% in group I, 82.6% in group II) had been continuously free of bladder relapses; on the other hand, 11.1% of the patients had experienced superficial relapse (9% in group I, 13% in group II) and 8.89% of the patients had muscle-invasive relapse (13.6% in group I, 4.3% is group II). These results were comparable with that reported by Zapatero *et al*. (34) where 6% of the complete responders developed superficial relapse and 9% developed muscle-invasive relapse. Kaufman *et al*. [23] in the RTOG 99-06, reported that 20% of the complete responders developed bladder relapses (superficial and muscle-invasive relapse) at 24 months follow-up. Tester *et al*. [19] in the RTOG 85-12, found that 17% of the complete responders experienced superficial relapse and 10.7% experienced muscle-invasive relapse at 36 months follow-up. Regional relapse occurred in 8.8% of the patients in the present study. Both groups were comparable as regard the incidence of regional relapse during the 24 month of follow-up, (7.1% in group I, 10.3% in group II).

In the present study, the patients were seen weekly during the chemoradiotherapy course to assess the incidence of acute treatment related toxicities. In general, the treatment was well tolerated and acute toxicities were transient and easily managed by symptomatic treatment. As regards the acute genitourinary (GU) toxicity during the induction phase, most of the patients (93.3% in group I and 86.7% in group II) had grade-1–grade-2 toxicity, while grade-3 toxicity was seen in 3.3% of patients in group I and 6.7% of patients in group II. There was no grade-4 toxicity. This was comparable with what was reported by Kaufman *et al* [20] where 4% of the patients had grade-3 acute genitourinary toxicity, with no grade-4 GY toxicity. The incidence of grade-1 and grade-2 acute gastrointestinal (GIT) toxicity during the induction phase were significantly higher in group I (86.7%) compared to group II (16.7%). This may be explained by the smaller field of radiotherapy and subsequently the limited bowel volume in group II. None of the patients in group II developed grade-3 or grade-4 acute GIT toxicity, while 6.7% of patients in group I experienced grade-3 GIT toxicity. The incidence of grade-3 acute GIT toxicity in group I of the present study (6.7%) was lower than that reported by Kaufman *et al* [20] where the incidence of acute grade-3 GIT toxicity was 15% (in the study of Kaufman, the patients are treated with whole pelvis in the induction phase similar to the patients in group I of the present study). The reason for the difference in the incidence rate may be due to difference in the sample size (80 patients in the study of Kaufman versus 30 patients only in group I of the present study).

In the current study, 80% of the patients in group I and 86.7% of patients in group II received adjuvant chemotherapy. Generally, the adjuvant chemotherapy was tolerated with neutropenia, nausea, vomiting, and fatigability being the most troublesome toxicities in both groups. As regards the late genitouninary toxicity, 21.7% of patients in group I and 25% of patients in group II developed grade-1 or grade-2 late toxicity, there was no grade-3 or grade-4 late urinary toxicity, and none of the patients required cystectomy as a treatment for late toxicity. Late gastrointestinal toxicity was more common in group I, with 8.7% of patients in group I having grade-I or grade-2 late GIT toxicity compared to 4.2% of patients in group II; however, this was not statistically significant. There was no grade-3 or grade-4 toxicity in both groups. Kaufman *et al* [20] had reported that 24% of the patients had grade-1 late toxicity (GU and GIT) and 12% had grade-2 toxicity (GU and GIT), 4% grade 3 and 2% grade 4 (only GU). No grade 3 or greater late GIT toxicity. The more toxicities observed in the study of Kaufman may be due to longer period of follow-up (5 years) compared to the present study (2 years).

In the current study, the 2-year overall survival was 75% in group I and 79.3% in group II, this was comparable to Kaufman *et al* [20] who reported that overall survival at 24 months was 73%, and comparable to Zapatero *et al* [21] where the overall survival at 36 months was 76%, also comparable to Lin *et al* [17] who reported 76.7% 3 years overall survival.

## Conclusion

To this end, we have shown that the concept of lymph node omission in bladder preservation protocol has not affected the overall survival of muscle invasive bladder cancer patients. Therefore, the results of this phase-II trial which included 60 patients encourage proceeding with future phase III trials with more patients.

## Figures and Tables

**Table 1.** Clinic-pathological characteristics of the studied groups.

Characteristics	Group I (*n* = 30) No. %		Group II (*n* = 30) No. %		Total (*n* = 60) No. %		Significance
**Age groups (years)**							
<55 55-60 >60	8 8 14	26.7 26.7 46.7	11 6 13	36.7 20.0 43.3	19 14 27	31.7 23.3 45.0	X^2^ = 0.80 *P* = 0.672
**Sex**							
Male Female	26 4	86.7 13.3	28 2	93.3 6.7	54 6	90.0 10.0	^FE^*P* = 0.671
**Stage of tumor**							
T2a T2b T3	6 18 6	20.0 60.0 20.0	9 17 4	30.0 56.7 13.3	15 35 10	25.0 58.3 16.7	X^2^ = 1.03 ^MC^*P* = 0.598
**Extent of TURBT**							
Complete Incomplete	23 7	76.7 23.3	25 5	83.3 16.7	48 12	80.0 20.0	X^2^ = 0.42 *P* = 0.519
**WHO performance status**							
1 2	25 5	83.3 16.7	25 5	83.3 16.7	50 10	83.3 16.7	X^2^ = 0.0 *P* = 1.0
**Smoking**							
Non smoker Mild to moderate Smoker Heavy smoker	12 13 5	40.0 43.3 16.7	10 14 6	33.3 46.7 20.0	22 38 11	36.7 63.3 36.7	X^2^ = 0.31 *P* = 0.857
**Bilharzias**							
Bilharzial Non bilharzial	23 7	76.7 23.3	19 11	63.3 36.7	42 18	70.0 30.0	X^2^ = 1.27 *P* = 0.259
**Presenting symptoms**							
Hematuria Hematuria and dysuria Hematuria, dysuria and frequency	19 6 5	63.3 20.0 16.7	22 5 3	73.3 16.7 10.0	41 11 8	68.3 18.3 13.3	X^2^ = 0.81 ^MC^*P* = 0.667
**Site of tumor**							
Right lateral wall Left lateral wall Anterior wall Dome or posterior wall Multiple sites	10 8 5 3 4	33.3 26.7 16.7 10.0 13.3	9 13 3 2 3	30.0 43.3 10.0 6.7 10.0	19 21 8 5 7	31.7 35.0 13.3 8.4 11.7	X^2^ = 2.09 ^MC^*P* = 0.719
**Size of tumor**							
<3 3 – 5 > 5	6 21 3	20.0 70.0 10.0	4 22 4	13.3 73.3 13.3	10 43 7	16.7 71.7 11.7	X^2^ = 0.570 ^MC^*P* = 0.753
**Multiplicity**							
Solitary tumor Multiple tumors	26 4	86.7 13.3	27 3	90.0 10.0	53 7	88.3 11.7	^FE^*P* = 1.0

X^2^ **Chi-Square test**

^MC^*P* **Monte Carlo corrected P-value**

^FE^*P* **Fisher’s Exact test**

**Table 2.** Initial response in the two studied groups.

Initial response	Group I (*n* = 30)		Group II (*n* = 30)		Total (*n* = 60)		Significance
	No.	%	No.	%	No.	%	
Complete	22	73.3	23	76.7	45	75.0	X^2^ = 0.09 *P* = 0.766
Incomplete	8	26.7	7	23.3	15	25.0	
***X*^2^: Chi-Square test.**							

**Table 3.** Incidence of regional relapse and distant metastasis in both groups.

Relapse and metastasis	Group I (*n* = 28)		Group II (*n* = 29)		Total (*n* = 57)		Significance
	No.	%	No.	%	No.	%	
**Regional relapse**							^FE^*P* = 1.0
No regional relapse	26	92.9	26	89.7	52	91.2	
Regional relapse	2	7.1	3	10.3	5	8.8	
**Distant metastasis**							^FE^*P* = 0.729
No metastasis	23	82.1	25	86.2	48	84.2	
Metastasis	5	17.9	4	13.8	9	15.8	
**^FE^*P*: Fisher’s Exact test.**							

**Table 4.** Acute toxicity during induction phase in both groups.

Acute toxicity	Group I (*n* = 30)		Group II (*n* = 30)		Total (*n* = 60)		Significance
	No.	%	No.	%	No.	%	
**1- Urinary toxicities**							
Absent	1	3.3	2	6.7	3	5.0	^FE^*P* = 1.0
Present	29	96.7	28	93.3	57	95.0	
g1–g2	28	93.3	26	86.7	54	90.0	
g3–g4	1	3.3	2	6.7	3	5.0	
**2- GIT toxicities**							
Absent	2	6.7	25	83.3	27	65.0	X^2^ = 35.62
Present	28	93.3	5	16.7	33	55.0	*P* < 0.0001*
g1–g2	26	86.7	5	16.7	31	51.7	
g3–g4	2	6.7	0	0.0	2	3.3	

*X*^2^ **Chi-Square test.**

^FE^*P* **Fisher’s exact test.**

* **Statistically significant at P ≤ 0.05.**

**Table 5.** Acute toxicity during consolidation phase in both groups.

Acute toxicity	Group I (*n* = 23)		Group II (*n* = 24)		Total (*n* = 47)		Significance
	No.	%	No.	%	No.	%	
**1- Urinary toxicities**							
Absent	2	8.7	4	16.7	6	12.8	^FE^*P* = 0.666
Present^	21	91.3	20	83.3	41	87.2	
**2- GIT toxicities**							
Absent	20	87.0	22	91.7	42	89.4	^FE^*P* = 0.666
Present^	3	13.0	2	8.3	5	10.6	

^ **All cases with toxicity were g1–g2.**

^FE^*P* Fisher’s exact test.

**Table 6.** Late toxicity in both groups.

Late toxicity	Group I (*n* = 23)		Group II (*n* = 24)		Total (*n* = 47)		Significance
	No.	%	No.	%	No.	%	
**1- Urinary toxicities**							
Absent	18	78.3	18	75.0	36	76.6	*χ*^2^ = 0.070
Present^	5	21.7	6	25.0	11	23.4	*P* = 0.792
**2- GIT toxicities**							
Absent	21	91.3	23	95.8	44	93.6	^FE^P = 0.609
Present^	2	8.7	1	4.2	3	6.4	

^ **All cases with toxicity were g1–g2.**

*X*^2^ **Chi-Square test.**

^FE^*P* **Fisher’s exact test.**
